# Cecal retroflexion is infrequently performed in routine practice and the retroflexed view is of poor quality

**DOI:** 10.1186/s12876-021-01877-4

**Published:** 2021-07-31

**Authors:** Rajesh N. Keswani, Charles J. Kahi, Mark Benson, Andrew J. Gawron, Tonya R. Kaltenbach, Rena H. Yadlapati, Dyanna L. Gregory, Anna Duloy

**Affiliations:** 1 Department of Gastroenterology and Hepatology, Northwestern University, 676 N. St. Clair, Suite 1400, Chicago, IL 60611 USA; 2 Department of Gastroenterology, Indiana University Medical Center, Indianapolis, USA; 3 Department of Gastroenterology and Hepatology, School of Medicine and Public Health, University of Wisconsin, Madison, USA; 4 Department of Gastroenterology, University of Utah, Salt Lake City, USA; 5 Department of Gastroenterology, University of California, San Francisco, USA; 6 Department of Gastroenterology, University of Colorado, Boulder, USA; 7 Division of Gastroenterology, University of California, San Diego School of Medicine, La Jolla, USA

**Keywords:** Retroflexion, Quality, Right colon

## Abstract

**Background:**

As right colon polyps are challenging to detect, a retroflexed view of right colon (RV) may be useful. However, cecal retroflexion (CR) without a RV to the hepatic flexure (HF) is inadequate. We aimed to determine the frequency of CR and quality of the RV in routine practice.

**Methods:**

This prospective observational study performed at an academic medical center assessed colonoscopy inspection technique of endoscopists who had performed ≥ 100 annual screening colonoscopies. We video recorded ≥ 28 screening/surveillance colonoscopies per endoscopist and randomly evaluated 7 videos per endoscopist. Six gastroenterologists blindly reviewed the videos to determine if CR was performed and HF withdrawal time (cecum to HF time, excluding ileal/polypectomy time).

**Results:**

Reviewers assessed 119 colonoscopies performed by 17 endoscopists. The median HF withdrawal time was 3 min and 46 s. CR was performed in 31% of colonoscopies. CR frequency varied between endoscopists with 9 never performing CR and 2 performing CR in all colonoscopies. When performed, nearly half (43%) of RVs did not extend to the HF with median RV duration of 16 s (IQR 9–30 s). Three polyps were identified in the RV (polyp detection rate of 8.1%), all identified prior to a forward view.

**Conclusions:**

CR is performed infrequently in routine practice. When CR is performed, the RV is of low quality with a very short inspection duration and insufficient ascending colon examination. Further education is required to educate endoscopists in optimal technique to improve overall colonoscopy quality.

## Background

Identification of neoplastic polyps during colonoscopy is integral to colorectal cancer (CRC) prevention. However, the quality of inspection during colonoscopy varies between endoscopists. When a colonoscopy is performed by an endoscopist who spends more time inspecting the colon for polyps (i.e., longer withdrawal time), their patients are less likely to develop interval CRC [1]. Similarly, patients who undergo a colonoscopy by endoscopists who detect more polyps are less likely to die of interval CRC [2]. In order to reduce the incidence of CRC, a cornerstone of colonoscopy quality improvement has been improving the ability of endoscopists to detect colon polyps.

Interval CRCs—cancers found within 3–5 years of a colonoscopy—are more likely to occur in the right colon [3]. While this may, in part, be related to the biology of right-sided colon lesions, these polyps are also more difficult to detect as they are more likely to be non-polypoid and “hidden” between deep colonic folds. Because right colon polyps are challenging to detect, multiple modalities have been suggested to improve their detection, including novel technology [4], multiple views of the right colon, and performing cecal retroflexion (“CR”) to obtain a retroflexed view of the right colon from the cecum (“RV”) [5]. While novel technology, including mucosal exposure devices and enhanced endoscopes, hold promise, they also incur an additional cost to colonoscopy. In contrast, a detailed examination of the right colon—either via two views of the right colon in forward view *or* one view in forward view and RV—may be sufficient to detect most polyps. In clinical trials, these strategies appear to be equivalent [5, 6].

Despite research supporting multiple views of the right colon, there is no data on how the right colon is examined in usual practice. To be effective, a RV should be performed to the hepatic flexure (HF) while circumferentially examining behind folds. However, in a prospective study evaluating the quality of colonoscopy inspection, we anecdotally noted that a RV was often performed in a brief, abbreviated manner [7]. Thus, endoscopists may be falsely reassured that they are performing an additional satisfactory second view of the right colon.

We hypothesized that the RV in clinical practice may differ from its performance in clinical trials. Thus, the primary aim of this study was to determine, in a diverse group of colonoscopists, the quality of the RV in usual practice. Specifically, we aimed to determine: (1) how often two views of the right colon are performed; (2) how frequently a second RV view of the right colon is performed, and (3) the extent and duration of RV of the ascending colon.

## Methods

### Study design and setting

We conducted a prospective observational study of colonoscopists performing screening and surveillance colonoscopy at a single urban academic medical center from October 3, 2016 to November 11, 2016. The Northwestern University Institutional Review Board approved the study (Institutional Review Board No. STU00203769, approval date September 8, 2016). Colonoscopists included in the study provided written informed consent.

Only colonoscopists who had performed 100 or more annual screening colonoscopies over a 2-year period preceding study onset were included. Over a 6-week period (October 3, 2016–November 11, 2016), study investigators prospectively recorded ≥ 28 de-identified screening or surveillance colonoscopies per colonoscopist. Colonoscopies performed for diagnostic indications, inflammatory bowel disease, or a personal history of a polyposis syndrome or cancer were excluded, as were colonoscopies with a Boston Bowel Preparation Score < 6. Finally, we also excluded colonoscopies performed with trainee participation.

Video recordings were obtained utilizing a portable high-definition digital video recorder (Sony HVO-500MD, Sony) attached to the digital endoscope processor. Prior to initiating recording, both patient and physician identifiers were removed. Although colonoscopists were aware of the recorders being set up in the procedure rooms, they were not informed of when they were specifically being recorded over the study period or why they were being recorded.

Seven videos per colonoscopist were randomly selected using a random number generator. Colonoscopy videos were evaluated by 6 U.S. gastroenterologists (R.Y., M.B., A.G., C.K., T.K., R.K.) with previous expertise in colonoscopy quality (“colonoscopy raters”). None of the six reviewers were included as one of the seventeen colonoscopists being evaluated.

### Video review and definitions

**Fig. 1:**
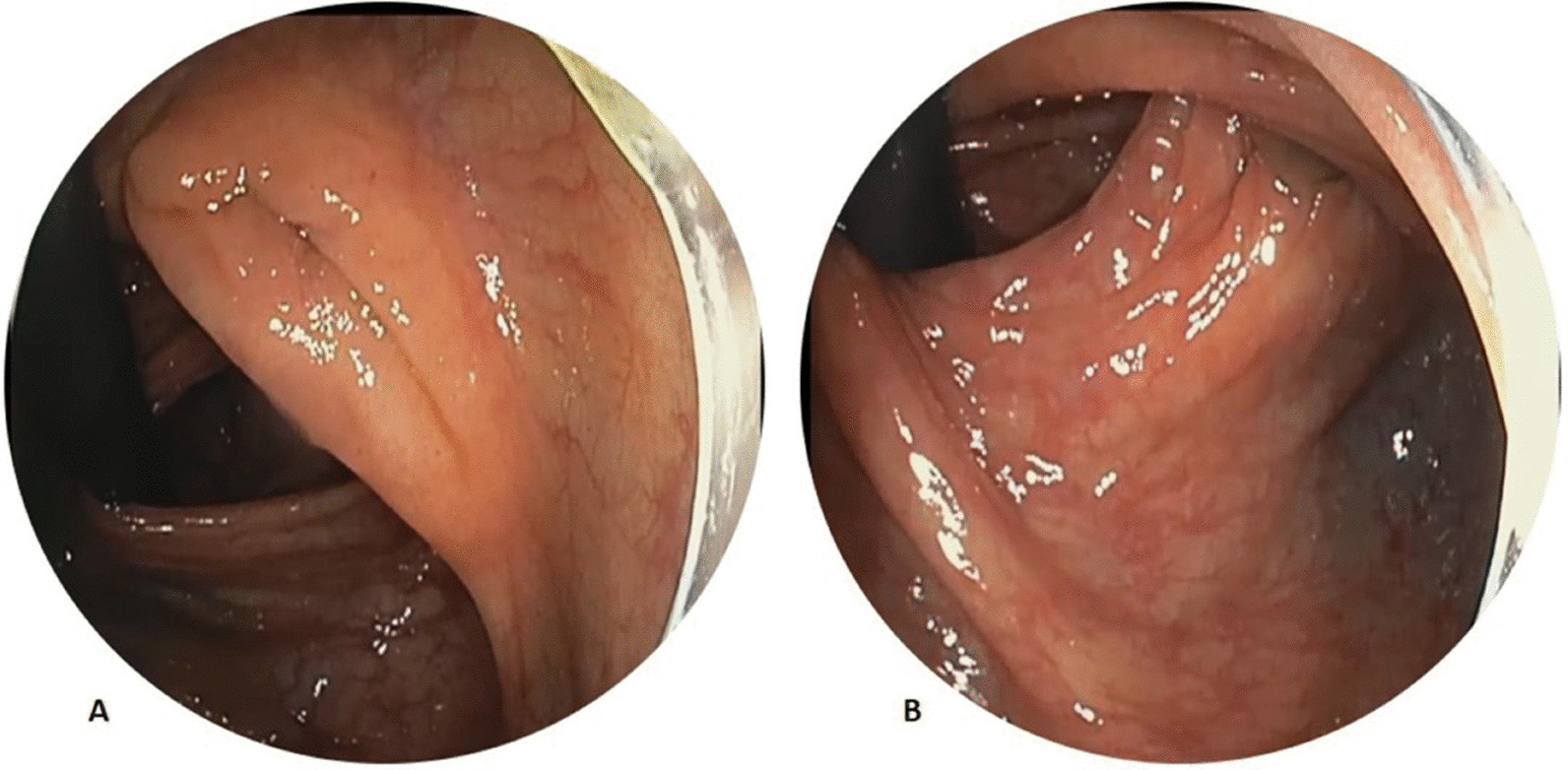
Cecal retroflexion (CR) with two different retroflexed views of the right colon (RV). **a** RV is just showing the ileocecal valve but is not extending to the level of the hepatic flexure (incomplete exam of right colon). **b** RV to the level of the hepatic flexure (complete exam of right colon)

Each of the colonoscopy raters individually and blindly reviewed recorded colonoscopy videos to determine: the number of complete examinations—either in forward view or retroflexed view—of the right colon (cecum to HF); hepatic flexure withdrawal time; whether a CR was performed; and the duration and extent of the RV. CR was defined as any successful retroflexion of the colonoscope within the cecum, regardless of the extent of the retroflexed exam (Fig. 1). The RV refers to the examination of the colon that occurs after CR is performed. A complete view of the right colon was defined as examination of the colon from the cecum to the hepatic flexure (in forward or RV). Thus, CR with a brief RV without examination to the level of the hepatic flexure was counted as CR being performed but not as a complete RV. The extent of the RV was documented (e.g., to mid-ascending colon, hepatic flexure, or transverse colon) as was the duration. Hepatic flexure withdrawal time was defined as the total time inspecting the colon from the cecum to hepatic flexure (combining all forward and all retroflexed views), excluding ileal/polypectomy time.

### Study outcomes

The primary study outcomes were the frequency of CR and quality of RV in usual practice.

### Statistical analysis

A complete case analysis was performed, we did not anticipate any missing data, and all analyses were planned a priori. *P* values < 0.05 were considered statistically significant. SAS version 9.4 (SAS Institute, Cary, NC) was used for all main statistical analyses. IBM SPSS Statistics 24 (IBM, Armonk, NY) was used for inter-rater reliability analysis.

## Results

Seventeen colonoscopists (16 gastroenterologists and 1 colorectal surgeon) met study inclusion criteria (≥ 100 annual screening colonoscopies) and provided informed consent. These 17 endoscopists performed a median of 482 annual screening colonoscopies (IQR 250–543) in the year preceding this study. The median historical WT was 11.1 (Interquartile Range [IQR], 8.7–14.0) minutes. The median ADR was 38% (IQR, 31–44%) and Serrated Polyp Detection Rate (SDR) was 10% (IQR, 8–13%). Seven videos per colonoscopist (119 videos total) were randomly selected for manual review.

### Right colon examination

At least two *complete* examinations of the right colon were performed in approximately one-quarter of colonoscopies (n = 31; 26.1%; Table 1). Among these 31 colonoscopies with two complete examinations of the right colon, 11 underwent a single examination in the forward view and a second complete examination of the right colon in the RV; 10 underwent ≥ 2 examinations in the forward view and an additional complete examination in the RV; and 10 patients underwent ≥ 2 examinations in the forward view without any complete examinations in the RV.

**Table 1:** Examination of the right colon (n = 119 colonoscopies)

CR performed	37 (31.1%)
Complete RV (to hepatic flexure) performed after CR	21 (56.8%)
Duration of RV, median [IQR]	16 s [9–30]
Two complete views of the right colon performed	31 (26.1%)
Single forward view exam and single RV exam	11 (35.5%)
≥ 2 Forward view exams and single RV exam	10 (32.2%)
≥ 2 Forward view exams without any RV exam	10 (32.2%)

In total, CR was performed in one-third of colonoscopies (31%; n = 37). However, the retroflexed exam *did not* extend to the HF in nearly half (43%) of these cases (i.e., incomplete RV). Only 2 colonoscopies underwent a RV beyond the hepatic flexure. The frequency with which CR (incomplete or complete) was performed varied between endoscopists with 9/17 colonoscopists never performing a CR and 2/17 performing a CR in all colonoscopies. For the two colonoscopists who always performed CR, the RV was to the level of the hepatic flexure in a majority (71.4%) of cases.

### Hepatic flexure withdrawal time

The median HF withdrawal time (defined as the total time spent inspecting the colon from the cecum to hepatic flexure, excluding polypectomy time, including all forward and retroflexed views) among all colonoscopies was 3 min and 46 s. The median HF withdrawal time in those colonoscopies with at least two complete examinations of the right colon was 5 min and 52 s.

The median RV duration (excluding polypectomy time) for all exams in which CR was performed was 16 s (IQR 9–30 s). As expected, the RV duration in exams that extended to the HF or beyond (complete RV) was significantly longer than in those exams that with an incomplete RV (28 vs. 11 s, *p* < 0.0001). The RV comprised a small proportion (8%) of total hepatic flexure withdrawal time in those exams in which retroflexion was performed.

Three polyps were identified in the RV (polyp detection rate of 8.1%). However, all 3 of these polyps identified in the RV were identified prior to a forward exam of the right colon.

## Discussion

In this study of 17 screening colonoscopists, we found that cecal retroflexion was performed in a minority (31%) of procedures with 9 colonoscopists never performing CR and 2 performing CR in all colonoscopies. Notably, even when CR was performed, nearly half (43%) of the retroflexed views did not extend to the HF (incomplete RV). Furthermore, the median RV duration (excluding polypectomy) was 16 s. In total, this data suggests that the CR is performed infrequently and the associated RV is of low quality.

Due to prior studies suggesting that colonoscopy does not protect against right-sided colon cancers [8], there has been increasing interest in improving the ability to identify polyps that may be hidden behind larger ascending colon folds. One method to potentially identify these polyps in the right colon is to perform CR—wherein the colonoscope looks back upon itself from the cecum to examine the ascending colon in a retroflexed view. However, since the more robust description of this technique in 2003[9], there has been conflicting data on the utility of this technique with most data suggesting that either method to view the right colon twice—either both in forward view or with the second view being a RV—is equivalent; however, it is clear that two view of the right colon are superior to a single view [10].

Despite a large amount of data comparing techniques utilized to examine the right colon, there is no data on what occurs in usual practice. In this study, we showed that only one-quarter of colonoscopies underwent two complete examinations of the right colon. Furthermore, when a RV was obtained via CR, it was often a brief evaluation of the ascending colon without a careful examination to the level of the hepatic flexure. This data importantly adds to the literature that shows how the effectiveness of an intervention in clinical practice may vary from its efficacy in a clinical trial [11]. In this case, endoscopists incorrectly consider a limited (both in extent and duration) RV as adequate. In contrast, in clinical trials the RV was standardized to examination to the level of the hepatic flexure [5]. As a result, after a low-quality RV, colonoscopists may be falsely reassured that they have performed an adequate second look in the right colon. This may further explain why the RV in our study had a low detection rate of neoplastic polyps.

There are important limitations to this study. We were only able to evaluate 7 screening colonoscopies per colonoscopist. Thus, there may be additional practice variations that we did not identify due to the smaller sample size. Furthermore, it is possible that physician practice of evaluating the ascending colon has evolved over time with accumulating data; this requires additional study. Finally, these findings represents the practice of endoscopists at a single center and external validation of our findings is required.

It is important to put our findings in the context of the now robust literature examining the methods to reduce polyp miss rate in the right colon. It is clear that two *full* views of the right colon, compared to one, reduces the miss rate of right colon neoplasia. However, we found that only 26.1% of colonoscopies had two complete views of the right colon, suggesting that further education is needed to ensure that all colonoscopists are performing high-quality colonoscopy. Furthermore, a second view either in the forward view or RV are equivalent. Our data suggests that colonoscopists performing CR with a second RV might be falsely reassured that they are performing a ‘second view’ despite their very limited RV extent and duration. Thus, further education is needed regarding the optimal method to view the right colon to reduce polyp miss rates.

## Conclusions

In summary, a minority of screening and surveillance colonoscopies undergo two evaluations of the right colon as currently recommended. In part, this may be due to our observation that when CR is performed, it is of short duration and inadequate extent. Further education on the optimal technique to evaluate the right colon after CR is needed.

## Data Availability

The datasets generated and/or analysed during the current study are not publicly available due to sensitivity of individual quality metrics data but are available from the corresponding author on reasonable request.

## Abbreviations

CRC: Colorectal cancer
IQR: Interquartile range
CR: Cecal retroflexion
HF: Hepatic flexure
RV: Retroflexed view of the right colon
SDR: Serrated polyp detection rate
